# Abdominal girth and vertebral column length can adjust spinal anesthesia for lower limb surgery, a prospective, observational study

**DOI:** 10.1186/s12871-016-0184-3

**Published:** 2016-03-24

**Authors:** Qing-he Zhou, Bo Zhu, Chang-na Wei, Min Yan

**Affiliations:** 1 Affiliation: Department of Anesthesiology, Second Affiliated Hospital, School of Medicine, Zhejiang University, Hangzhou, Zhejiang Province China; 2 Affiliation: Department of Anesthesiology, Second Affiliated Hospital of Jiaxing University, Jiaxing, Zhejiang Province China

**Keywords:** Anaesthetic techniques, Subarachnoid, Anaesthetics local, Bupivacaine, Apinal cord, Sensory block

## Abstract

**Background:**

Studies have shown that abdominal girth and vertebral column length have high predictive value for spinal spread after administering a dose of plain bupivacaine. we designed a study to identify the specific correlations between abdominal girth, vertebral column length and a 0.5 % dosage of plain bupivacaine, which should provide a minimum upper block level (T_12_) and a suitable upper block level (T_10_) for lower limb surgeries.

**Methods:**

A suitable dose of 0.5 % plain bupivacaine was administered intrathecally between the L_3_ and L_4_ vertebrae for lower limb surgeries. If the upper cephalad spread of the patient by loss of pinprick discrimination was T_12_ or T_10_, the patient was enrolled in this study. Five patient variables and intrathecal plain bupivacaine dose were recorded. Linear regression and multiple regression analyses were performed.

**Results:**

Totals of 111 patients and 121 patients who lost pinprick discrimination at T_12_ and T_10_, respectively, were analyzed in this study. Linear regression analysis showed that only abdominal girth and plain bupivacaine dose were strongly correlated (*r* =−0.827 for T_12,_ *r* = −0.806 for T_10;_ both *p* < 0.0001). Multiple linear regression analysis showed that both abdominal girth and vertebral column length were the key determinants of plain bupivacaine dose (both *p* < 0.0001). R^2^ was 0.874 and 0.860 for the loss of pinprick discrimination at T_12_ and T_10_, respectively.

**Conclusions:**

Our data indicated that vertebral column length and abdominal girth were strongly correlated with the dosage of intrathecal plain bupivacaine for the loss of pinprick discrimination at T_12_ and T_10_. The two regression equations were Y_T12_ = 3.547 + 0.045X_1_-0.044X_2_ and Y_T10_ = 3.848 + 0.047X_1_- 0.046X_2_ (Y, 0.5 % plain bupivacaine volume; X_1_, vertebral column length;and *X*_2_, abdominal girth), which can accurately predict the minimum and suitable intrathecal bupivacaine dose for lower limb surgery to a great extent, separately.

## Background

Plain bupivacaine spinal anesthesia is a conventional technique [1–5]. However, the spread of intrathecal plain bupivacaine is highly unpredictable [6]. Previous studies have proven that at least 25 factors could affect the spread of spinal anesthesia [7–9]. Lumbosacral cerebrospinal fluid volume was found to be the primary determinant that affect spinal cephalad spread [10], lumbosacral cerebrospinal fluid pressure was also an important factor that affects spinal cephalad spread [11], but those informations had little practical value because they were inconvenient to obtain. Clinically, patient’s age, height, weight, and body mass index (BMI), have commonly been considered when determining the dose of bupivacaine. However, previous studies have reported that these variables were only weakly to moderately correlated with and had little predictive value for the spread of spinal anesthesia [1, 3, 12–14].

Our previous study showed that abdominal girth and vertebral column length were highly predictive of the spread of spinal anesthesia after a given dose of plain bupivacaine [15]. However, the results did not suggest a specific dose of bupivacaine for an individual. T_12_ spinal spread level was nearly the minimum block level for eliminating tourniquet pain and T_10_ spinal spread levels could provide satisfactory analgesia with almost no hemodynamic effects for lower limb surgery. Plain 0.5 % bupivacaine has frequently been used for spinal anesthesia [1, 3, 13, 16]. Therefore, we designed a prospective, observational study to identify the specific correlations between abdominal girth, vertebral column length and a 0.5 % dosage of plain bupivacaine, which should provide a minimum upper block level (T_12_) and a suitable upper block level (T_10_) for lower limb surgeries.

## Methods

### Ethics

This study was approved by the Ethical Committee of the Second Affiliated Hospital of Jiaxing University (Ethical Committee number CZJE 201311; Chairperson Professor Li-qin Jiang) on 11 February 2013. All patients provided prior written informed consent.

### Patients

A total of 692 patients were enrolled in this study from February 2013 to March 2014, and the following inclusion criteria were applied: ASA physical status I and II, 19–60 years old, and were scheduled for lower limb fractures surgery, hip arthroplasty, knee arthroplasty, knee arthroscopy, great saphenous vein surgery and so on. Patients with contraindications for spinal anesthesia, diabetes, rheumatoid arthritis, spinal canal stenosis, a history of spinal anesthesia or spinal surgery, pregnant patients and patients with language disorders were excluded.

### Intervention

All patients fasted for 8–10 h before surgery. After the patients entered the operating room, intravenous access was established, and Ringer’s lactate 10 ml/kg was preloaded before anesthesia. Noninvasive arterial pressure was recorded every 5 min; electrocardiography and pulse oximetry were continuously monitored for all patients. The patients were placed in supine position, and at the level of the umbilicus, the abdominal girth was measured at the end of expiration. Before spinal anesthesia was administered, the patient was placed in the right lateral decubitus position, with spinal column flexion. During spinal anesthesia, the L3-4 interspace was confirmed by ultrasound imaging, a 25-gauge Quincke spinal needle was inserted, and a midline approach was adopted, with the orifice pointing toward the cephalad. We set T_10_ spinal block level as the target level, and a dose of 0.5 % plain bupivacaine that the anesthesiologist deemed suitable was injected intrathecally with room temperature at a speed of approximately 2 ml in 10 s when free flow of the cerebrospinal fluid was obtained. After the procedure, the patient was placed supine position immediately. The spinal anesthesia spread was assessed using an 18-gauge sharp needle every 10 min in both midclavicular lines till 60 min after the intrathecal injection. At each level, three pin touches were employed for the loss of pinprick discrimination. If the ultimate spinal anesthesia level of loss of pinprick discrimination in both midclavicular lines was T_12_ or T_10_ in 60 min after the intrathecal injection, the patient was picked out to analyze in our study, and the vertebral column length was measured from the C7 vertebra to the sacral hiatus, with the patient placed supine on the operation table. The sacral hiatus and C7 vertebra were both confirmed by radiographic imaging.

Two anesthetists participated in the study. All anesthesia procedures and patient management were performed by the same anesthetist, and assessment of the spinal anesthesia spreads were performed by another anesthetist, who was unaware of the bupivacaine dose used in each patient. Patients^,^age, height, weight, vertebral column length abdominal girth and 0.5 % bupivacaine dose were recorded.

### Statistical analysis

The sample size was determined as described in reference 15. If the anticipated effect size was 0.15, the desired statistical power level was 0.8, the predictors were 5, and the minimum required sample size was 91, with a probability level of 0.05. SPSS software (SPSS Inc., Chicago, IL, USA), version 19.0, was used to analyze the data. Linear regression analysis was performed to determine the correlations between the spinal anesthesia -induced loss of pinprick discrimination at T_12_ or T_10_ and patients^,^ age, weight, height, abdominal girth, and vertebral column length. Multiple regression analysis was performed to test the combined linear contributions of patients^,^age, height, weight, vertebral column length and abdominal girth to the bupivacaine dose for T_10_ or T_12_ loss of pinprick discrimination. During multiple regression analysis, stepwise selection was performed, and the predictors were removed from the analysis if they were not significantly correlated with the dependent variable. R^2^ was the coefficient of determination. A *p* value < 0.05 was considered to be statistically significant.

## Results

**Fig. 1:**
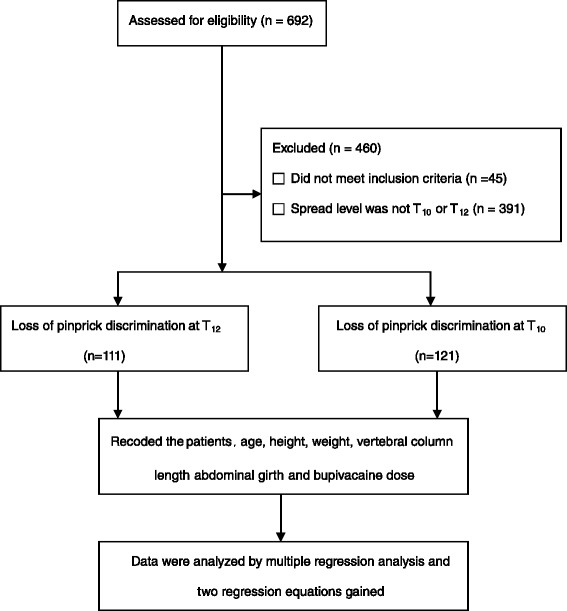
Consort flowchart for the intrathecal plain bupivacaine dose can be predicted by abdominal girth and vertebral column length

Of 692 potential participants, 45 failed to meet the inclusion criteria,391 patients had spread levels other than T_10_ or T_12_ (the maximum block level was T_4_, the minimal block level was L_2_), and 24 were excluded for other reasons. The remaining 232 patients (111 patients with loss of pinprick discrimination at T_12_, 121 patients with loss of pinprick discrimination at T_10_) were analysed in this study (Fig. 1). There were no significant differences in the variables between those patients with loss of pinprick discrimination at T_12_ and at T_10_ (*p* > 0.05) (Table 1).

**Table 1:** Patient variables and bupivacaine doses

	Loss of pinprick discrimination at T_12_ (*n* = 111)	Loss of pinprick discrimination at T_10_ (*n* = 121)
ASA status (I/II)	82/29	95/26
Sex, male/female	71/40	83/38
Age, y	44.6 (11.7)	44.9 (11.9)
Height, cm	165.2(8.3)	165.9(7.3)
Weight, kg	64.3 (10.9)	67.9 (11.3)
Vertebral column length, cm	65.2(4.9)	65.2(4.4)
Abdominal girth, cm	78.1(9.9)	81.5(8.8)
0.5 % plain bupivacaine volume, ml	3.04(0.51)	3.18(0.44)

**Fig. 2:**
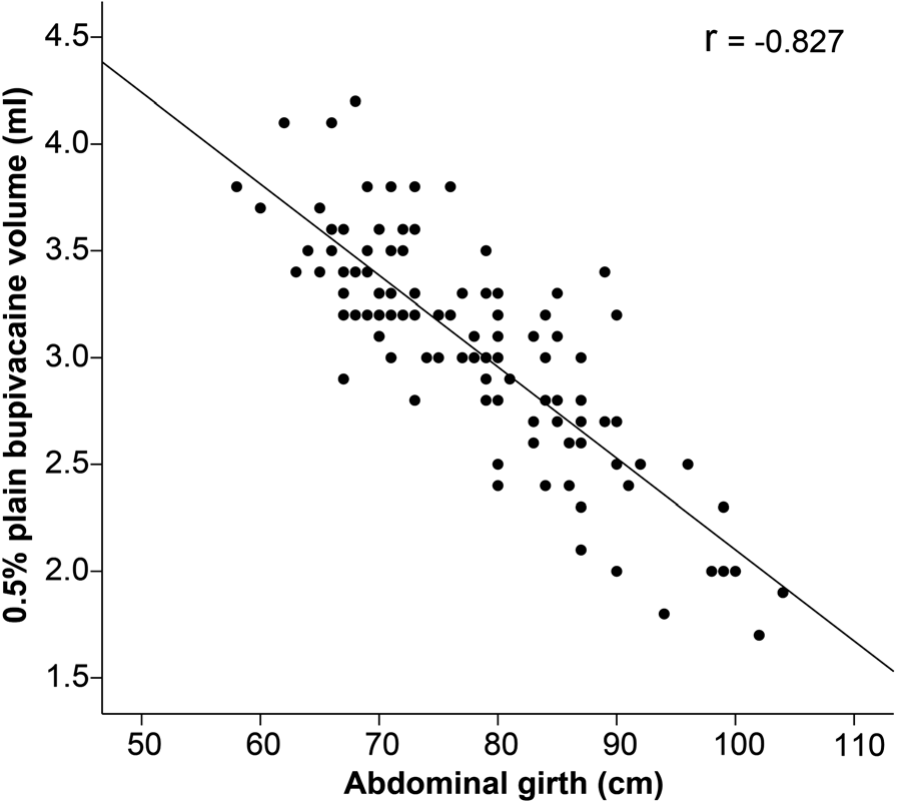
Linear regression analysis of abdominal girth and 0.5 % plain bupivacaine volume for loss of pinprick discrimination at T_12_. *r* = -0.827, *p* < 0.0001. *r*: correlation coefficient

**Fig. 3:**
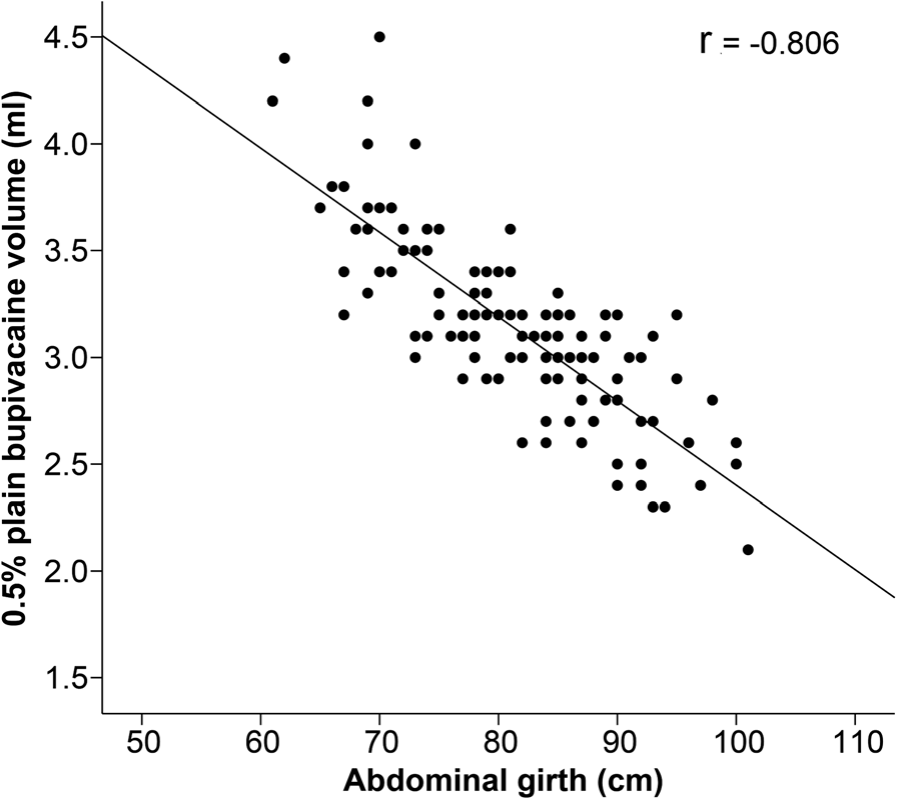
Linear regression analysis of abdominal girth and 0.5 % plain bupivacaine volume for loss of pinprick discrimination at T_10_. *r* = -0.806, *p* < 0.0001. *r*: correlation coefficient

Linear regression analysis showed that there were significant univariate correlations between height, weight, abdominal girth, and vertebral column length and the 0.5 % plain bupivacaine volume for loss of pinprick discrimination at T_12_ or T_10_ (all *p* < 0.0324). However, only abdominal girth and 0.5 % plain bupivacaine volume were strongly correlated (*r* = −0.827 for T_12,_ *r* = −0.806 for T_10;_ *p* < 0.000) (Table 2; Figs. 2 and 3).

**Table 2:** The relationship of patient variables with the bupivacaine dose for loss of pinprick discrimination at T_12_ and T_10_

Patient characteristics	0.5 % plain bupivacaine volume for loss of pinprick discrimination at T_12_		0.5 % plain bupivacaine volume for loss of pinprick discrimination at T_10_	
	*r*	*p* value	*r*	*p* value
Age	−0.335	<0.001	−0.362	<0.001
Height	0.367	<0.001	0.195	0.0324
Weight	−0.494	<0.001	−0.460	<0.001
Vertebral column length	0.383	<0.001	0.203	0.0257
Abdominal girth	−0.827	<0.001	−0.806	<0.001

*r:* correlation coefficient

Multiple linear regression analysis showed that abdominal girth and vertebral column length were the key determinants of 0.5 % plain bupivacaine volume (both *p* < 0.0001), while age, weight, and height could be omitted without changing the results (all *p* > 0.164, all 95 % confidence intervals < 0.267) (Table 3). R^2^ was 0.874 for the loss of pinprick discrimination at T_12_ and 0.860 for the loss of pinprick discrimination at T_10_.

**Table 3:** The combined linear regression of patient variables with bupivacaine dose for loss of pinprick discrimination at T_12_ and T_10_

Patient characteristics	0.5 % plain bupivacaine volume for loss of pinprick discrimination at T_12_				0.5 % plain bupivacaine volume for loss of pinprick discrimination at T_10_			
	*b*	*p* value	*95 % CI*		*b*	*p* value	*95 % CI*	
			lower limit	upper limit			lower limit	upper limit
Age	0.016	0.667	−0.140	0.226	0.027	0.725	−0.100	0.232
Height	0.020	0.717	−0.122	0.267	0.084	0.164	−0.098	0.245
Weight	−0.062	0.422	−0.331	0.107	0.090	0.200	−0.119	0.231
Abdominal girth	−0.044	<0.001	−0.777	−0.563	−0.046	<0.001	−0.861	−0.713
Vertebral column length	0.045	<0.001	0.431	0.726	0.047	<0.001	0.399	0.648

*b:* regression coefficient; 95 % CI: 95 % confidence interval for the partial correlation coefficients

The regression equations between the 0.5 % plain bupivacaine volume and vertebral column length, abdominal girth for loss of pinprick discrimination at T_12_ and at T_10_ were Y_T12_ = 3.547 + 0.045X_1_-0.044X_2_ and Y_T10_ = 3.848 + 0.047X_1_- 0.046X_2_, respectively (Y, 0.5 % plain bupivacaine volume; X_1_, vertebral column length;and *X*_2_, abdominal girth).

## Discussion

The most important finding of the present study was that vertebral column length and abdominal girth were strongly correlated with the dosage of intrathecal plain bupivacaine for the loss of pinprick discrimination at T_12_ and T_10_. This finding indicated that the requirement of intrathecal plain bupivacaine dose could be largely predicted by vertebral column length and abdominal girth for lower limb surgeries.

A previous study showed that the volume of lumbosacral cerebrospinal fluid was the primary determinant of sensory block level during spinal anesthesia [10], and there was broad variation in the volume of lumbosacral cerebrospinal fluid between individuals, which decreased following an increase in intra-abdominal pressure [11]. Greater abdominal girth was associated with a more notable increase in the intra-abdominal pressure [17]. Logic might suggest that there would be a smaller volume of lumbosacral cerebrospinal fluid in patients with shorter vertebral column lengths. Thus, to a great extent, abdominal girth and vertebral column length could accurately predict the dosage of intrathecal plain bupivacaine for T_12_ and T_10_ block level.

Many studies have investigated the effects of a patient’s age [13], height [1, 12], weight and BMI [1, 12, 14] on the spread of plain bupivacaine spinal anesthesia, but the results have been contradictory, or the variables have demonstrated to be weakly correlated with the spread of spinal anesthesia. Using three-dimensional magnetic resonance imaging, Sullivan and his colleagues [18] further proved that only weak correlations existed between the volume of lumbosacral cerebrospinal fluid and height, weight or BMI. In our previous and current studies, multiple linear regression analysis also showed that among the five predictors, age, weight and height had low value for predicting the dosage of intrathecal bupivacaine for the level of anticipant spinal anesthesia.

To date, some studies have been performed to investigate the optimal dose of intrathecal plain bupivacaine for certain surgeries [5, 19]. However, these studies have only demonstrated the range of the intrathecal bupivacaine (e.g., ED_50_ or ED_95_) but not the optimal individual dose.^5^ Indeed, the ED_50_ or ED_95_ values could provide some guidance for using local anesthetics, but even if low doses of intrathecal plain bupivacaine were used, hypotension could still occurred because of excessive cephalad spread [19].

In previous studies, up-down sequential analysis [20] and logistic regression analyses [5] have been used to determine the optional dose of local anesthetics. Neither method could determine the optimal dose for an individual because the individual response to intrathecal local anesthesia varies greatly. In the current study, multiple linear regression analysis was performed to investigate the combined linear contribution of five patient variables to the volume of 0.5 % plain bupivacaine for loss of pinprick discrimination at T_12_ and T_10_, then the regression equations between the 0.5 % plain bupivacaine volume and vertebral column length, abdominal girth were obtained. The R^2^ for loss of pinprick discrimination at both T_12_ and T_10_ was approximately 0.9, indicating that the regression equation could accurately predict the dosage of specific intrathecal bupivacaine for T_12_ or T_10_ spinal spread in an individual.

In this study, reverse thoughts were used. We set T_10_ spinal block level as the target level, and the bupivacaine dose administered was based on the experience of the anesthetist. No matter how much the bupivacaine dose were used, there were many possibilities that some patients^,^ spinal upper block level would be T_12_ or T_10_. When the sample size, based on spinal spread for loss of pinprick discrimination at T_12_ and T_10_, reached 91 respectively could the study be terminated. Finally, 111 patients with loss of pinprick discrimination at T_12_ and 121 patients with loss of pinprick discrimination at T_10_ were enrolled in our study. 391 patients with loss of pinprick discrimination for other spinal levels were not analyzed.

Reynolds F. [21] advised avoiding spinal injection above L_3_ to decrease the risk of spinal cord trauma. Therefore, the L_3_-L_4_ interspace is usually the targeted intervertebral needle insertion site during spinal anesthesia, and it was adopted in our study. The accuracy of the regression equation for the dosage of intrathecal bupivacaine and vertebral column length and abdominal girth for T_10_ and T_12_ spinal spread levels should be confirmed in the future with larger samples. The subjects of this study were 19 to 60 years old; therefore, the accuracy of the regression equation for patients of other ages should also be confirmed.

## Conclusion

In conclusion, our data indicated that vertebral column length and abdominal girth were strongly correlated with the dosage of intrathecal plain bupivacaine for the loss of pinprick discrimination at T_12_ and T_10_. The two regression equations were Y_T12_ = 3.547 + 0.045X_1_-0.044X_2_ and Y_T10_ = 3.848 + 0.047X_1_- 0.046X_2_ (Y, 0.5 % plain bupivacaine volume; X_1_, vertebral column length;and *X*_2_, abdominal girth), which can accurately predict the minimum and suitable intrathecal bupivacaine dose for lower limb surgeries to a great extent separately.

## Abbreviations

ASA: American Standards Association
BMI: body mass index
ED_50_: median effective dose
ED_95_: 95 % effective dose
